# Melt-Spinning of an Intrinsically Flame-Retardant Polyacrylonitrile Copolymer

**DOI:** 10.3390/ma13214826

**Published:** 2020-10-28

**Authors:** Simon König, Philipp Kreis, Christian Herbert, Andreas Wego, Mark Steinmann, Dongren Wang, Erik Frank, Michael R. Buchmeiser

**Affiliations:** 1 German Institutes of Textile and Fiber Research, Körschtalstr. 26, D-73770 Denkendorf, Germany; simon.koenig@ditf.de (S.K.); philipp.kreis@ditf.de (P.K.); mark.steinmann@ditf.de (M.S.); erik.frank@ditf.de (E.F.); 2 Institute of Polymer Chemistry, University of Stuttgart, Pfaffenwaldring 55, D-70569 Stuttgart, Germany; dongren.wang@ipoc.uni-stuttgart.de; 3 Dralon GmbH, Chempark Dormagen, Postfach 10 04 85, 41522 Dormagen, Germany; christian.herbert@dralon.com (C.H.); andreas.wego@dralon.com (A.W.)

**Keywords:** flame retardation, polyacrylonitrile, PAN, melt spinning

## Abstract

Poly(acrylonitrile) (PAN) fibers have two essential drawbacks: they are usually processed by solution-spinning, which is inferior to melt spinning in terms of productivity and costs, and they are flammable in air. Here, we report on the synthesis and melt-spinning of an intrinsically flame-retardant PAN-copolymer with phosphorus-containing dimethylphosphonomethyl acrylate (DPA) as primary comonomer. Furthermore, the copolymerization parameters of the aqueous suspension polymerization of acrylonitrile (AN) and DPA were determined applying both the Fineman and Ross and Kelen and Tüdõs methods. For flame retardancy and melt-spinning tests, multiple PAN copolymers with different amounts of DPA and, in some cases, methyl acrylate (MA) have been synthesized. One of the synthesized PAN-copolymers has been melt-spun with propylene carbonate (PC) as plasticizer; the resulting PAN-fibers had a tenacity of 195 ± 40 MPa and a Young’s modulus of 5.2 ± 0.7 GPa. The flame-retardant properties have been determined by Limiting Oxygen Index (LOI) flame tests. The LOI value of the melt-spinnable PAN was 25.1; it therefore meets the flame retardancy criteria for many applications. In short, the reported method shows that the disadvantage of high comonomer content necessary for flame retardation can be turned into an advantage by enabling melt spinning.

## 1. Introduction

Poly(acrylonitrile) (PAN) used for textile fibers is mostly polymerized by precipitation polymerization and then solution-spun from polar solvents. PAN-based fibers are widely used for clothing, home textiles and technical textiles [1,2,3,4,5,6,7]. In all these fields of application, flame-retardant textiles are increasingly in demand by consumers or are required by law [8]. 

PAN has a “Limiting Oxygen Index” (LOI) of about 17–18 determined according to DIN EN ISO 4589-1, i.e., it starts to burn at oxygen concentrations of the surrounding atmosphere ≥17–18 vol% (e.g., air), if it is exposed to a flame for longer than a second [9]. For PAN to be called flame-retardant in air according to the DIN-norm, its LOI value has to be larger than 20.8. However, as a safety net, for most applications LOI values ≥25 are aimed for [10,11].

To increase the LOI of a polymer, one has to add flame retardants. These can be introduced as additives, as comonomers, by chemical modification or by coating. Additives and coatings are usually the cheapest way to provide a fiber with flame retardant properties [12] however, both are not a good choice with regard to a long-term flame-retardant effect. In contrast to chemical modifications, they tend to migrate or get washed out of the fiber [11]. It seems therefore prudent to introduce flame retardants by copolymerization or chemical modification.

The most powerful chemical modification known for PAN is a post-spinning-treatment with hydrazine [8,13,14,15,16]. Such fibers reach very high LOI values of up to 40 [16,17]. The mechanism of the reaction and the mechanism of the flame retardancy are not fully understood, but the decisive functional group appears to be a bishydrazidine [13]. However, due to the toxicity, carcinogenicity, explosiveness, corrosiveness and flammability of hydrazine and the resulting strict regulations and necessary safety precautions that exist in most countries, this method is barely used in industry [18]. Analogous methods in which hydrazine is substituted by less toxic diamines are being researched, but have not yet resulted in comparable LOI values [19].

An example for flame retardant comonomers of acrylonitrile are halogenated comonomers like vinyl chloride, vinylidene chloride and vinyl bromide. They are used in concentrations of 30–50 mol%. The resulting intrinsically flame-retardant fibers reach LOI values of up to 30 and are known as “modacrylic fibers” [12,20]. However, HCl or HBr as well as various environmentally harmful and toxic halogenated hydrocarbons are released during burning of modacrylic fibers [21,22]. Phosphorus-containing comonomers are a promising alternative [9,23,24,25,26,27,28,29,30]. The phosphorus in these comonomers is usually present in the oxidation states +III or +V in the form of phosphonates or phosphates. The precise structure of the phosphorus-containing comonomer seems to play a subordinate role for the flame-retardant effect; more important is the mass fraction of phosphorus contained in the polymer. To reach an LOI value of 25, about 3 wt% phosphorus in the PAN are required [29,30]. Phosphorus-containing flame retardants take effect in both, the flame and the condensed phase. In the flame, various phosphorus-containing radicals (PO_2_, PO, HPO) suppress the radical chain reactions by recombination [31,32]. In the condensed phase they accelerate the cyclization of PAN. In addition, they may form a protective, non-flammable layer of poly(HPO_3_) [33].

A promising phosphorus-containing comonomer for PAN is dimethylphosphonomethyl acrylate (DPA) (16.0 wt% P), which has already been proven to copolymerize readily with acrylonitrile (AN) and allow for wet-spinning, resulting in fibers with LOI values of ≥ 25 at ca. 3 wt% phosphorus [30]. In case of DPA, 3 wt% of phosphorus in the polymer translates into a comonomer content of 18.8 wt% or 6.3 mol%, respectively. This is very close to the range of ca. 7–20 mol% required for (plasticized) melt spinning of PAN [34,35,36,37,38,39,40,41,42,43]. Recently, we published on the plasticized melt-spinning of a flammable PAN using propylene carbonate (PC) as plasticizer [38]. Here, we report on the synthesis and PC-plasticized melt-spinning of an intrinsically flame-retardant PAN-PDPA-(PMA)-copolymer. The reported copolymerization solves two problems at the same time: it ensures flame retardation and enables plasticized melt spinning.

## 2. Materials and Methods

### 2.1. Chemicals

Acrylonitrile (>99%), acryloyl chloride (96%), dichloromethane (99.9%), methanol (99%), methyl acrylate (>99%), potassium carbonate (99%), sodium persulfate (98%), and triethyl amine (99%) were purchased from abcr GmbH (Karlsruhe, Germany). Sodium metabisulfite (99%), propylene carbonate (99%), iron (II) sulfate hydrate (>99%), and aluminum sulfate hydrate (98–102%) were purchased from Alfa Aesar (Kandel, Germany). Sulfuric acid (96%) was purchased by Acros Organics (Morris Plains, NJ, USA). Dimethyl phosphite (98%) was purchased from Sigma Aldrich (Darmstadt, Germany). All chemicals were used as received. 

### 2.2. Synthesis of Dimethyl (Hydroxymethl)phosphonate 

Dimethyl (hydroxymethl)phosphonate was synthesized according to El Asri et al. [44] Dimethyl phosphite (110.6 g, 1 mol) and paraformaldehyde (30.0 g, 1 mol) were dissolved in 330 mL methanol and cooled to 0 °C. K_2_CO_3_ (6.77 g 0.05 mol) was then added to the stirred mixture. The mixture was allowed to warm to room temperature and was stirred for four hours. The K_2_CO_3_ was then filtered off and the volatile components were removed at reduced pressure (1 ∙ 10^−2^ mbar). Yield: 98 g, 70%. ^1^H-NMR (400 MHz, CDCl_3_) *δ* = 4.28 (s, 1H), 3.91 (d, *J* = 6.0 Hz, 2H), 3.77 (d, *J* = 10.5 Hz, 6H) ppm. ^31^P-NMR (162 MHz, CDCl_3_) *δ* = 26.9 ppm. ^13^C-NMR (101 MHz, CDCl_3_) *δ* = 56.1 (d, *J* = 164 Hz), 53.2 (d, *J* = 7.1 Hz) ppm. HRMS (ESI): *m*/*z* calculated for C_3_H_10_O_4_P^+^: 141.0322. Found: 141.0319.

### 2.3. Synthesis of Dimethyl Phosphonomethylacrylate (DPA) 

Dimethyl phosphonomethylacrylate was synthesized according to Liepins et al. [25] Dimethyl (hydroxymethyl)phosphonate (67.23 g, 0.48 mol) was dissolved in CH_2_Cl_2_ (400 mL) and the solution was cooled to 0 °C. Within one hour, acryloyl chloride (47.79 g, 0.53 mol) dissolved in 100 mL CH_2_Cl_2_ was added. The reaction mixture was stirred for another two hours. The resulting triethylamine hydrochloride was filtered off and all volatile components were removed under reduced pressure. The raw product distilled at 83 °C and 1.7 ∙ 10^−2^ mbar. Yield: 40 g, 71%. ^1^H-NMR (400 MHz, CDCl_3_): *δ* = 6.49 (dd, *J* = 17.3, 1.3 Hz, 1H), 6.18 (dd, *J* = 17.3, 10.5 Hz, 1H), 5.93 (dd, *J* = 10.5, 1.3 Hz, 1H), 4.49 (d, *J* = 8.7 Hz, 2H), 3.82 (d, *J* = 10.8 Hz, 6H) ppm (Figure S1). ^31^P-NMR (162 MHz, CDCl_3_) δ = 21.57 (s) ppm (Figure S2). ^13^C-NMR (101 MHz, CDCl_3_) *δ* = 165.0 (d, *J* = 8.0 Hz), 132.5, 127.0, 56.0 (d, *J* = 169.1 Hz), 53.3 (d, *J* = 6.2 Hz) ppm. HRMS (ESI): *m*/*z* calculated for C_6_H_12_O_5_P^+^: 195.0428. Found: 195.0417.

### 2.4. Polymerizations

Aqueous precipitation polymerizations were carried out under an argon atmosphere at 56 °C in aqueous, acidic medium (0.03 molar aqueous sulfuric acid solution). After preheating the aqueous medium, the monomer mixture was added and stirred for one minute. The monomer concentration in the aqueous reaction mixture was 6.7 wt%. Then, the initiator was added. The initiator system consisted of sodium persulfate (PAN F1, F3, F4: 0.14 wt%; PAN F2, F5: 0.28 wt% with respect to the total reaction mixture), sodium metabisulfite (PAN F1, F3, F4: 0.35 wt%; PAN F2, F5: 0.7 wt% with respect to the total reaction mixture), catalytic amounts of iron (II) sulfate (ca. 1 ppm with respect to the total reaction mixture) and aluminum sulfate (50–100 ppm with respect to the total reaction mixture) dissolved in demineralized water (3.3 wt% with respect to the total reaction mixture). After adding the initiator to the stirred reaction mixture, the polymerization mixture was stirred for another hour. The polymer precipitated as a fine white powder which was filtered off, washed twice with water and methanol, respectively, and dried at 80 °C under atmospheric pressure. The resulting polymers and their properties are summarized in Table 1.

### 2.5. Calculation of the Copolymerization Parameters

The parameters *G* and *H*, which are being used in the graphic methods to determine the copolymerization parameters according to Fineman and Ross [45] and Kelen and Tüdõs [46,47] were defined as follows:

(1) $$G=\;\frac{\left[M_{\mathrm{DPA}}\right]}{\left[M_{\mathrm{AN}}\right]}\cdot \left(1-\frac{\left[m_{\mathrm{AN}}\right]}{\left[m_{\mathrm{DPA}}\right]}\right)$$

(2) $$H=\;\frac{{\left[M_{DPA}\right]}^{2}}{{\left[M_{AN}\right]}^{2}}\cdot \frac{\left[m_{AN}\right]}{\left[m_{DPA}\right]}$$

In these equations, *M_DPA_* and *M_AN_* are the molar amounts of DPA and AN in the educt mixture and *m_DPA_* and *m_AN_* are the molar amounts of AN and DPA in the copolymer. In the Fineman and Ross plot, the parameters *G* and *H* are plotted linearly according to Equation (3) to determine *r_DPA_* and *r_AN_*:

(3) $$G=r_{AN}H-r_{DPA}$$

For the inverted Fineman and Ross plot, *G* and *H* are plotted according to Equation (4):

(4) $$\frac{G}{H}=r_{DPA}\frac{1}{H}-r_{AN}$$

Kelen and Tüdõs extended the Fineman and Ross method with a parameter *α* to distribute the data points evenly (Equation (5)):

(5) $$\frac{G}{\alpha +H}=\left(r_{DPA}+\frac{r_{AN}}{\alpha }\right)\frac{H}{\alpha +H}-\frac{r_{AN}}{\alpha }$$

with:

(6) $$\alpha =\sqrt{H_{min}\cdot H_{max}}$$

### 2.6. Drying

In addition to drying at 80 °C under atmospheric pressure after polymerization, all polymers were dried under vacuum at 100 °C for 5 h prior to rheology measurements, spinning trials, and film preparation in a vacutherm VT 6130 M vacuum furnace (Heraeus Holding GmbH, Hanau, Germany) equipped with a rz-16 vacuum pump (Vacuubrand GmbH & Co. KG, Wertheim, Germany). The residual water content was determined by Karl Fischer titration, which was performed on an 899 Coulometer and an 885 Compact Oven SC (Methrom AG Zofingen, Schweiz) at 140 °C. The resulting water content was <0.1 wt%.

### 2.7. Film Preparation

PAN films were prepared by doctor blading and the immersion casting technique. First, a 24 wt% solution in DMSO was prepared for each PAN. Then, the viscous polymer solution was poured onto a glass plate and evenly distributed by a stainless-steel thin film applicator (doctor blade) with a gap height of 500 μm. The glass plate was then immersed in water as a coagulation bath. After 14 h, the coagulated polymer film was removed and dried at 80 °C for 2 h. Then, they were compressed by a heat press at 6 bar pressure and 90 °C. An example of the resulting polymer film is shown in Figure S3.

### 2.8. Melt Spinning

Fibers were melt-spun using a Haake MiniLab II twin-screw extruder (Thermo Fisher Scientific Inc., Waltham, MA, USA) with a capacity of ca. 7 mL. A monofilament spinning nozzle with a diameter of 0.5 mm was used. The fibers were wound on a Sahm winder (Georg Sahm GmbH, Eschwege, Germany).

### 2.9. Plasticizer Removal

Removal of propylene carbonate (PC) was achieved discontinuously by winding the fibers around a glass vial and then putting them in stirred demineralized water at 90 °C for 5 min. The residual PC-content was below 0.1 wt% determined by ^1^H-NMR.

### 2.10. Characterization

NMR spectra were measured on a Ultrashield 400 Plus instrument (Bruker Corporation, Billerica, MA, USA) at 303 K. (^1^H-NMR: 400 MHz, ^13^C-NMR: 101 MHz; external standard: tetramethylsilane). Spectra were analyzed using the MestReNova program (Mestrelab Research, Santiago de Compostela, Spain). Rheology was measured on a Physica MCR 501 (Anton Paar Group AG, Graz, Austria). Measurements were carried out with two parallel plates in a distance of 1 mm. For mechanical testing of the fibers, 20 single filament measurements of each sample were conducted on a Favimat tensiometer (Textechno Herbert Stein GmbH & Co. KG, Mönchengladbach, Germany). The clamping length was 25 mm, the test speed was 20 mm/min. Size exclusion chromatography (SEC) measurements were performed on *a 1260 Infinity* device from Agilent Technologies (Santa Clara, CA, USA) equipped with a Multi-Detector-Suite viscosimetry and refractive index detector. A 50 mm precolumn and a 300 mixed B column (Agilent Technologies) were used as stationary phases, DMAc containing 5 g/L LiBr was used as mobile phase. The column temperature was set to 50 °C, the flow rate was 0.75 L/min, the sample concentration was 2 mg/mL. Chromatograms were interpreted using conventional calibration against poly(methyl methacrylate) polymer standards in a molecular weight range of 102 < *M_n_* < 392,000 g/mol. Differential scanning calorimetry (DSC) measurements were carried out under air flow (20 mL/min) on a Q2000 differential scanning calorimeter (TA Instruments Inc., New Castle, DE, USA) applying a heating rate of 10 K/min. The sample mass was 2 mg. High resolution mass spectrometry was carried out on a Microtof Q mass spectrometer (Bruker Daltonics, Billerica, MA, USA). Elemental analysis for determining the phosphorus content was achieved via precipitation titration. 30 mg of a sample were dissolved in 10 mL of perchloric acid at 100 °C over two days. The solution was then titrated with aqueous lanthanum nitrate, resulting in the precipitation of lanthanum phosphate. A color change indicated the end of the titration, the phosphorus content was calculated from the consumed lanthanum nitrate. Limiting oxygen index (LOI) analyses were performed by burning PAN-films in a testing atmosphere with varying oxygen content in accordance to DIN EN ISO 4589-1 on an apparatus provided by Fire Testing Technology (East Grinstead, UK). A section of the film (5 cm × 15 cm) was used as the test specimen and clamped vertically in a holder. This holder was located inside a glass cylinder through which an oxygen-nitrogen mixture with a defined oxygen content was passed. The sample was ignited from above. By varying the oxygen content, the maximum oxygen concentration was determined at which the sample still extinguished by itself, which is the LOI.

## 3. Results and Discussion

### 3.1. Copolymerization Parameters of the Aqueous Precipitation Copolymerization of AN and DPA

**Scheme 1:**
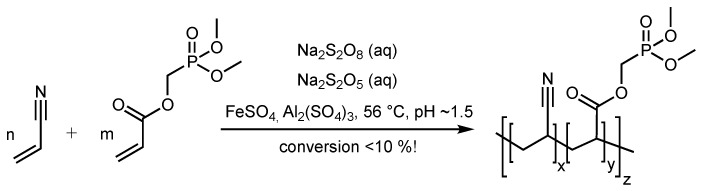
Synthesis of PAN-*co*-PDPA. For the synthesis of the copolymer samples for the determination of copolymerization parameters, conversions <10% were necessary.

In order to better understand the copolymerization of AN and DPA, their copolymerization parameters were determined. Monomers were copolymerized by precipitation polymerization as outlined in Scheme 1 in the molar ratios shown in Table 2.

Molar ratios of AN:DPA < 90:10 were not used since the resulting copolymers did not precipitate completely but partially dissolved in DPA. After about one minute of polymerization time, the target conversion of 5–10% was reached. All copolymers were checked for their molar composition by ^1^H-NMR in DMSO-d_6_.

**Figure 1:**
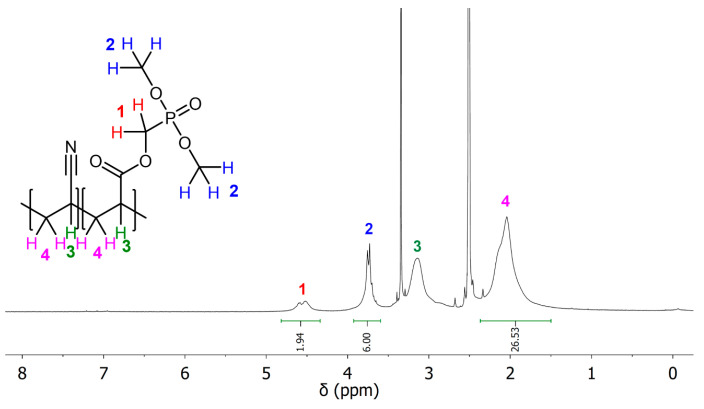
^1^H-NMR of a PAN with 10 mol% DPA in the reaction mixture and 7.5 mol% incorporated in the copolymer at a conversion of 6.3% (see Table 2), measured in DMSO-d_6_.

Figure 1 shows a representative ^1^H-NMR spectrum of a PAN-*co*-PDPA copolymer with all signals assigned. To determine the molar composition in the copolymer, the CH_2_-signals **4** in the backbone of the polymer chain were compared with the signals of the two CH_3_-groups **2** of the methyl esters of DPA in the copolymer. By integrating and comparing these two characteristic signals, the ratio of the two comonomer units in the copolymer was determined.

**Figure 2:**
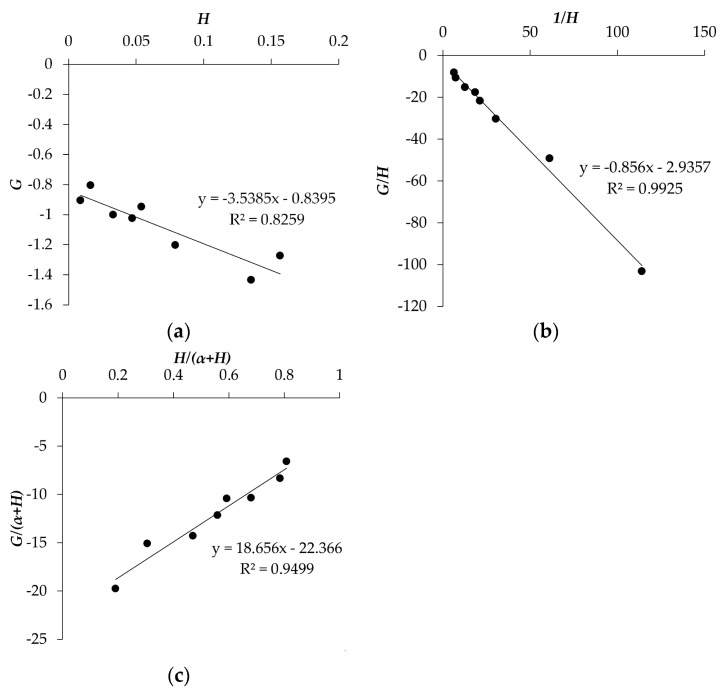
Graphic determination of *r_DPA_* and *r_AN_* for the aqueous precipitation copolymerization of AN and DPA at 56 °C. (**a**): Plot of G against H according to Fineman and Ross. (**b**): Plot of *G/H* against *1/H* according to Fineman and Ross (inverted). (**c**): Plot of $\frac{G}{\alpha +H}$ against $\frac{H}{\alpha +H}$ according to Kelen and Tüdõs with *α* = 0.04.

Subsequently, three different methods were used to determine the copolymerization parameters *r_DPA_* and *r_AN_*: The Fineman and Ross plot, [45] the inverted Fineman and Ross plot, [45] as well as the Kelen and Tüdõs plot [46,47]. A disadvantage of the Fineman and Ross plots is that the data points are not evenly distributed and that data points resulting from particularly high or particularly low proportion of one of the comonomers are clearly overrated (see Figure 2b). The Kelen and Tüdõs method solved this by extending the Fineman and Ross method with a parameter *α* for a more even distribution of the data points. 

Figure 2 shows the plots for the precipitation copolymerization of AN with DPA; the resulting copolymerization parameters *r_DPA_* and *r_AN_* are summarized in Table 3. The determined copolymerization parameters were in a similar range for all three methods. The average value for *r_DPA_* was 0.84 ± 0.05, the one for *r_AN_* was 3.4 ± 1.2. The determined copolymerization parameters imply that all polymer radicals react preferentially with AN, regardless of whether the reactive polymer radical at the chain end is derived from DPA or AN. According to these copolymerization parameters, which are both still close to unity, a slight compositional gradient in the copolymer composition must be expected to occur over the entire course of the copolymerization. At low conversion, the AN content in the copolymers will be higher due to their preferential incorporation, whereas at high conversion the DPA content in the copolymers will increase due to the shift in the AN:DPA ratio of the yet unreacted monomers. However, in view of the poor copolymerization propensity of other phosphorus-containing vinyl compounds [28,30] DPA seems to be a good choice. 

### 3.2. Synthesis and Properties of PAN-copolymers

Next, five PAN-based copolymers, PAN F1–F5 (Table 1) were synthesized. In view of the copolymerization parameters, which indeed suggest the formation of a gradient copolymer (*vide supra*), the molar amount of DPA required to obtain a melt-spinnable PAN was anticipated to be higher than for copolymers prepared from comonomers with copolymerization parameters with AN closer to unity, e.g., MA (*r_AN_* = 0.7–1.0, *r_MA_* = 0.7–1.5) [38,48,49,50]. Furthermore, such higher DPA-concentration was anticipated to be necessary as the statistical distribution of the comonomer is crucial for its effectiveness as an internal plasticizer, the more since polymer chains with a high AN-content have an increased tendency to gel [43]. Unfortunately, the amount of DPA in the starting mixture turned out to be limited to <10 mol% for the aqueous precipitation polymerization setup used in this work, since higher DPA concentrations led to a partial dissolution of PAN in DPA, impeding proper precipitation. In order to overcome this limitation in comonomer concentration, MA was added as second comonomer leading to the terpolymers PAN F3–F5. In line with the copolymerization parameters, PANs F3–F5 contained less DPA in the polymer than in the starting mixture (feed).

Flame retardant properties can be determined by various standardized methods like the UL94 vertical flame test (DIN EN 60695-11-10), by microscale combustion calorimetry (ASTM D7309), or by the LOI vertical flame test (DIN EN ISO 4589-1). These different measurements all have advantages and limitations. They also suffer from poor intercorrelation amongst each other (*r^2^* usually ca. 0.4–0.7) [51]. This has to be kept in mind when interpreting the flammability data in this work, where only LOI-tests have been conducted.

PAN F1, PAN F2 and PAN F5 matched the targeted phosphorus content of > 3 wt% (Table 4). In order to check the LOI values of all PANs, films were produced from these PAN copolymers and then examined for flammability in flame tests. For this purpose, 24 wt% of each PAN was dissolved in DMSO. The viscous solutions were then distributed evenly with a thickness of 500 microns applying doctor blading; then they were immersion cast in demineralized water. Figure S3 shows such a film. Table 4 summarizes the LOI values of the films. In accordance with the literature, [23,24,28,29] terpolymers containing >3 wt% phosphorus had an LOI value >25. 

Additionally, both the *T_g_* and *T_deg_* values were determined by DSC in air; measurements are shown in Figure S4. *T_g_* was in the expected range of ca. 100 °C for all PANs. The onset temperature *T_deg_* of the exothermal cyclization and dehydrogenation reactions of PAN was in the range of 239–255 °C in air for PAN F1–F5 and lower than *T_deg_* of a PAN-homopolymer (*T_deg_* = 298 °C) [52] or a comparable PAN-copolymer with 8.1 mol% of MA (*T_deg_* = 291 °C) [38]. 

### 3.3. Rheology and Melt-spinning of a PAN-co-DPA Plasticized with PC

In this work, propylene carbonate (PC) was used as an external plasticizer in addition to DPA and MA as internal plasticizers, since PC is cheap, non-toxic and can be easily removed by washing with water [38]. Table 5 shows the rheological properties of PAN F1–F5 mixed with 22.5 wt% PC. 

**Figure 3:**
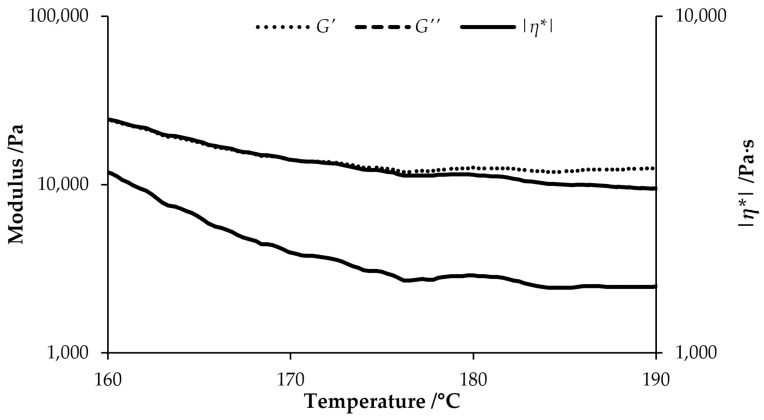
Temperature sweep from 150 to 190 °C of PAN F5 mixed with 22.5 Gew. % PC. *γ* = 0.5%, *ω =* 10 rad/s, heating rate = 1 K/min.

Oscillatory rheology temperature sweeps from 160 to 190 °C were measured. For melt spinning, a target viscosity of |η*| > 2000 Pa s was aimed for, ideally in the range of 200–1000 Pa s. Furthermore, the loss factor *tan δ* should be at least ≥ 1, indicating acceptable viscoelastic behavior of the PAN/PC mixture. While PAN F1–F4 were all outside the desired |η*| < 2000 Pa s and *tan δ* ≥ 1, PAN F5 met the criteria. Its temperature sweep measurement is shown in Figure 3. The value for |η*| of was <2000 Pa s in a temperature range of 170–190 °C. Its tan δ at 170 °C was 1 and decreased slightly to 0.97 at 175 °C. This can tentatively be attributed to minor PAN-degradation over time that overlays the measurement due to the slow heating rate of 1 K/min and the comparably low *T_deg_* of 239 °C. 

Melt-spinning of PAN F5 blended with 22.5 wt% PC was successfully carried out at 175 °C. A photograph of the resulting fibers is shown in Figure S10. In line with the comparably low *tan δ-*value, winding speeds up to 30 m/min were reached. The mechanical properties of the fiber before and after removal of the PC are summarized in Table 6.

Although the fibers had a comparably large diameter due to the low winding speeds, their tenacity after washing was almost 200 MPa, which is better than expected for such low speeds. Tenacity was approx. 10–50% lower than in commercial, flammable textile PAN-fibers, but comparable to hydrazine-modified flame-retardant PAN-fibers or modacrylic fibers [15,53,54]. Consequently, a melt-spinnable PAN with flame retardant properties and sufficient mechanical properties can indeed be regarded feasible.

## 4. Conclusions

The scope of this work was to prepare a flame retardant, melt-spinnable PAN. This was achieved with a flame-retardant PAN-terpolymer containing both, MA and DPA, as comonomers. The copolymer was melt-spun using PC as an external plasticizer, yielding fibers with tenacities slightly lower than in commercial PAN-fibers. In agreement with the literature, PAN-copolymers with >3 wt% phosphorus incorporated had a LOI value >25.

Copolymerization parameters of AN and DPA were determined to *r_DPA_* = 0.84 ± 0.05 and *r_AN_* = 3.4 ± 1.2, which indicates that a gradient in polymer composition is formed over the polymerization time.

## Figures and Tables

**Table 1:** Summary of molecular weights, phosphorus contents determined by elemental analysis and the amounts of DPA and MA of the synthesized PAN-copolymers.

PAN	*M_AN_* * /mol%	*M_DPA_* * /mol%	*M_MA_* * /mol%	P-content Calc. /wt%	P-content Found ** /wt%	*m_DPA_* *** /mol%	${\bar{M}}_{n}\;/g/\mathrm{mol}$	*Đ*	Yield /g	X **** /%
PAN F1	91.9	8.1	0	3.89	3.59	7.5	43 000	5.5	34	61
PAN F2	91.9	8.1	0	3.89	3.60	7.5	35 000	5.3	30	55
PAN F3	91.9	6.0	2.1	3	2.63	5.3	51 000	4.7	28	50
PAN F4	90.2	6.1	3.7	3	2.31	4.7	57 000	4.4	7.5	67
PAN F5	88	8.2	3.8	3.89	3.08	6.5	43 000	5.1	7.0	62

* Molar amount in the educt mixture determined by weight. ** Phosphorus content determined by elemental analysis. *** Molar amount in the polymer based on the phosphorus content as determined by elemental analysis. **** Conversion.

**Table 2:** Molar amounts of AN and DPA in the polymerization mixture and in the resulting polymers, which were synthesized for determining the copolymerization parameters in quantities of ca. 300 mg.

*M_AN_* */mol%	*M_DPA_* */mol%	*m_AN_* **/mol%	*m_DPA_* **/mol%	X ***/%
99	1	99	1.0	6.4
98	2	97.7	2.3	5.5
97	3	97.0	3.0	7.1
96	4	96.0	4.0	5.1
95	5	94.9	5.1	8.1
94	6	95.3	4.7	6.9
92	8	94.5	6.6	6.6
90	10	92.5	7.5	6.3

* Molar amount in the reaction mixture determined by weight. ** Phosphorus content determined by elemental analysis. ** Molar amount in the polymer based on ^1^H-NMR analysis according to Figure 1. *** Conversion.

**Table 3:** Summary of the copolymerization parameters *r_DPA_* and *r_AN_* resulting from the Fineman and Ross and Kelen and Tüdõs plots in Figure 2.

Method	*r_DPA_*	*r_AN_*
Fineman and Ross	0.84 ± 0.06	3.5 ± 0.7
Inverted Fineman and Ross	0.86 ± 0.03	2.9 ± 1.5
Kelen and Tüdõs	0.83 ± 0.06	3.7 ± 1.4
Average	0.84 ± 0.05	3.4 ± 1.2

**Table 4:** Summary of the P-content determined by elemental analysis, LOI values determined by flame tests of the PAN-films and the *T_g_* and *T_deg_* values determined by DSC (Figure S4).

PAN	P-content Found/wt%	LOI (Film)	*T_g_*/°C	*T_deg_*/°C
PAN F1	3.59	26.4	99	240
PAN F2	3.60	26.5	98	248
PAN F3	2.63	23.2	99	250
PAN F4	2.31	22.2	99	255
PAN F5	3.08	25.1	95	239

**Table 5:** Summary of *tan δ* and |η*| at 175 °C for mixtures of 22.5 wt% of PC and the synthesized PAN-*co*-PDPAs (temperature sweep measurements are shown in Figures S4–S7).

Sample	Possible Spinning Temperature/°C	*tan δ* at 170 °C	*tan δ* at 175 °C	|η*| at 170 °C/Pa⋅s	|η*| at 175 °C/Pa⋅s
PAN F1/PC	-	0.38	0.38	7600	7600
PAN F2/PC	-	0.34	0.32	7100	7800
PAN F3/PC	-	n.d.*	n.d.*	n.d.*	n.d.*
PAN F4/PC	-	0.47	0.42	11,600	12,000
PAN F5/PC	170(-175?)	1.0	0.97	1970	1740

* not determinable because the viscosity of the PAN/PC mixture was too high, so that it could not be compressed to the desired measuring gap of 1 mm.

**Table 6:** Mechanical properties of PAN F5 fibers spun at 30 m/min before and after discontinuous washing in water for plasticizer removal (stress-strain diagram are shown in Figures S9 and S10).

Plasticizer Removed	Diameter /μm	Elongation /%	Tenacity /cN/tex	Tenacity /MPa	Young’s Modulus /cN/tex	Young’s Modulus /GPa
no	49 ± 9	15.5 ± 2.5	9 ± 3	100 ± 40	80 ± 30	1.0 ± 0.4
yes	44 ± 13	19 ± 5	17 ± 4	195 ± 40	440 ± 60	5.2 ± 0.7

## Supplementary Materials

The following material is available online at https://www.mdpi.com/1996-1944/13/21/4826/s1, Figure S1: ^1^H-NMR spectrum of DPA after vacuum distillation, Figure S2: ^31^P NMR spectrum of DPA after vacuum distillation, Figure S3: Example of a PAN film made of PAN F3, Figure S4: DSC measurements in air of of PAN samples F1–F5 applying a heating rate of 10 °C/min, Figure S5: Temperature sweep from 150 to 190 °C of PAN sample F1 (8.1 mol% DPA, ${\bar{M}}_{n}$ = 43,000 g/mol, *Đ* = 5.5), mixed with 22.5 wt% PC. *γ* = 0.5%, *ω =* 10 rad/s, heating rate = 1 K/min, Figure S6: Temperature sweep from 150 to 190 °C of PAN sample F2 (8.1 mol% DPA, ${\bar{M}}_{n}$ = 35 000 g/mol, *Đ* = 5.3), mixed with 22.5 wt% PC. *γ* = 0.5%, *ω =* 10 rad/s, heating rate = 1 K/min, Figure S7: Temperature sweep from 150 to 190 °C of PAN sample F4 (6.1 mol% DPA, 3.7 mol% MA, ${\bar{M}}_{n}$ = 57,000 g/mol, *Đ* = 5.5), mixed with 22.5 wt% PC. *γ* = 0.5%, *ω =* 10 rad/s, heating rate = 1 K/min, Figure S8: Stress-strain diagram (tensile test) of PAN F5 fibers (8.2 mol% DPA, 3.8 mol% MA, ${\bar{M}}_{n}$ = 43,000 g/mol, *Đ* = 5.1) containing 22.5 wt% PC wound at a winding speed of 30 m/min, Figure S9: Stress-strain diagram (tensile test) of PAN F5 fibers spun (8.2 mol% DPA, 3.8 mol% MA, ${\bar{M}}_{n}$ = 43,000 g/mol, *Đ* = 5.1), spun with 22.5 wt% PC at a winding speed of 30 m/min. PC was washed out in demineralized water at 90 °C over 5 min, Figure S10: Photograph of PAN F5 (8.2 mol% DPA, 3.8 mol% MA, ${\bar{M}}_{n}$ = 43,000 g/mol, *Đ* = 5.1) fibers containing 22.5 wt% PC. Fibers were wound at a winding speed of 30 m/min.
