# Supplementary material for: Melt-Spinning of an Intrinsically Flame-Retardant Polyacrylonitrile Copolymer
Source: Materials (Basel). 2020 Oct 28;13(21):4826. doi: 10.3390/ma13214826 (PMC7663686; doi:10.3390/ma13214826)
Supplement: Supplementary file 1 [file materials-13-04826-s001.pdf]

Supplementary Materials

to

Melt-Spinning of an Intrinsically Flame-Retardant Polyacrylonitrile Copolymer

Simon König[1], Philipp Kreis[1], Christian Herbert[3], Andreas Wego[3], Mark Steinmann[1], Dongren Wang[2], Erik Frank[1], Michael R. Buchmeiser*[1,2]

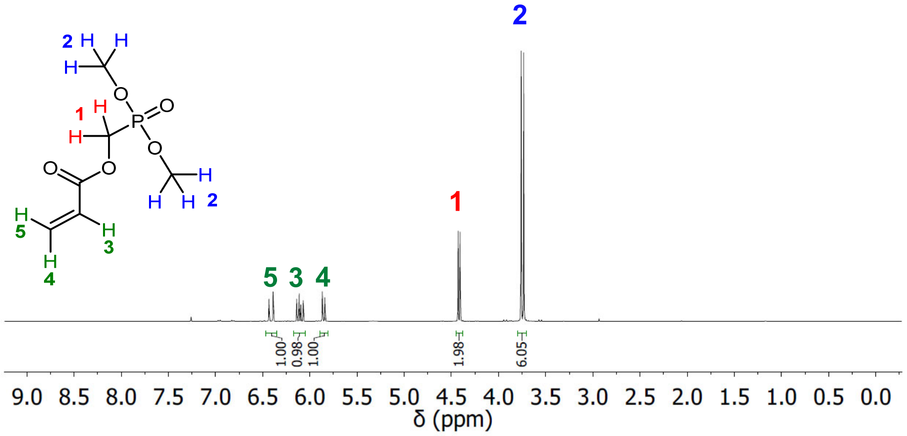

Figure S1. ^{1}H NMR spectrum of DPA after vacuum distillation.

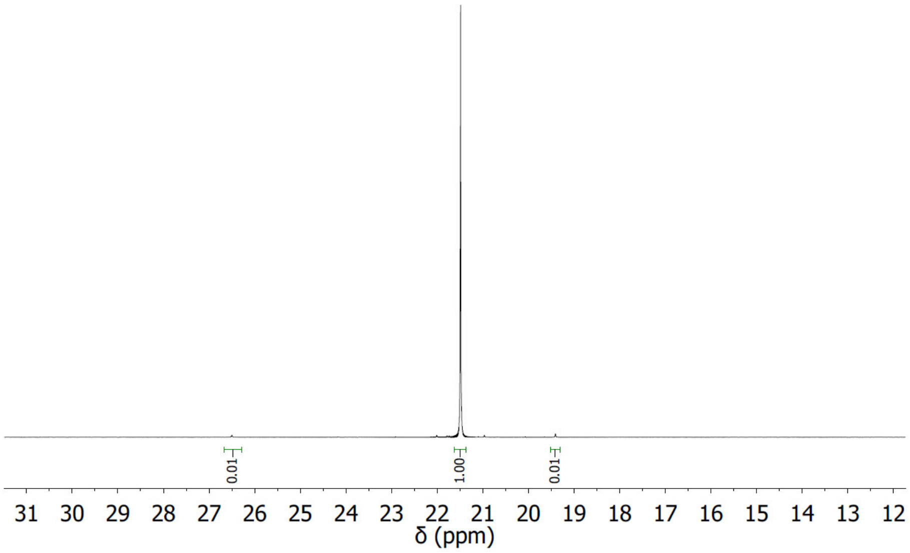

Figure S2. ^{31}P NMR spectrum of DPA after vacuum distillation.

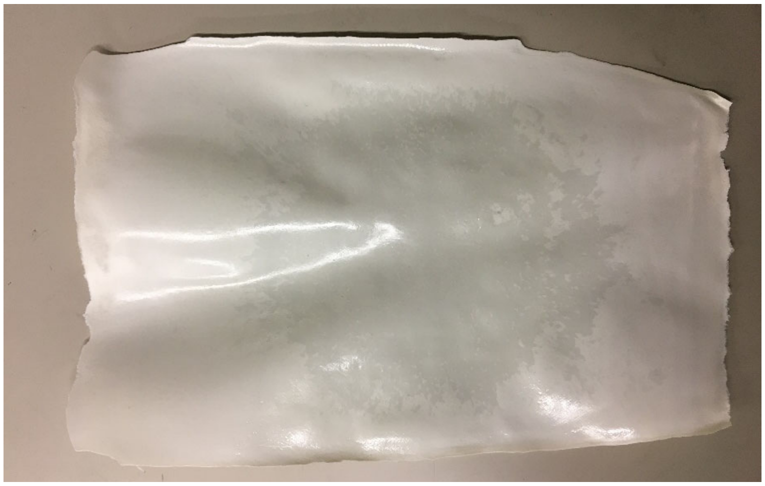

Figure S3. Example of a PAN film made of PAN F3.

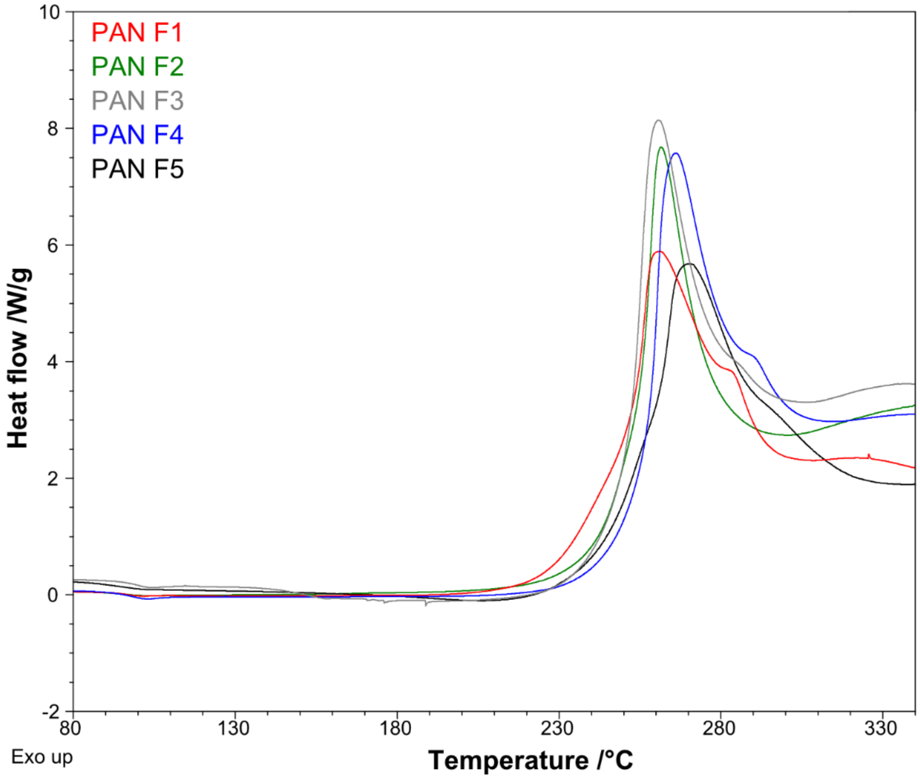

Figure S4. DSC measurements in air of PAN samples F1–F5 applying a heating rate of 10 °C/min.

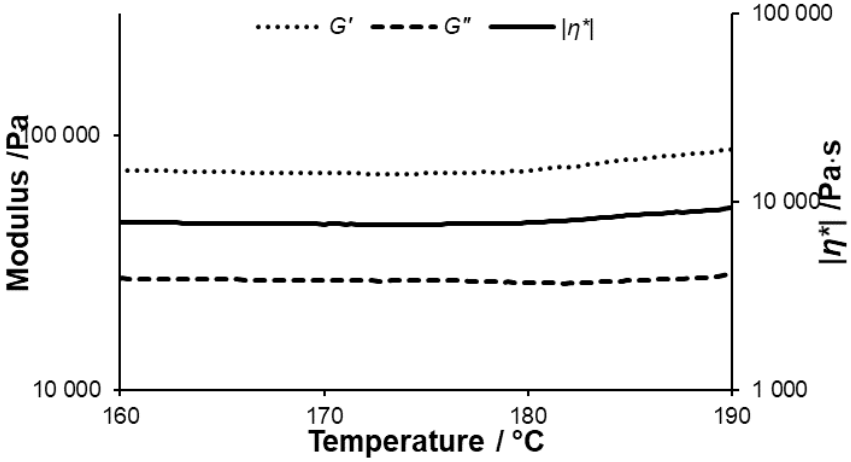

Figure 5. Temperature sweep from 150 to 190 °C of PAN sample F1 (8.1 mol-% DPA, $\overline{M}_n$ = 43, 000 g/mol, *Đ* = 5.5), mixed with 22.5 wt.-% PC. γ = 0.5 %, ω = 10 rad/s, heating rate = 1 K/min.

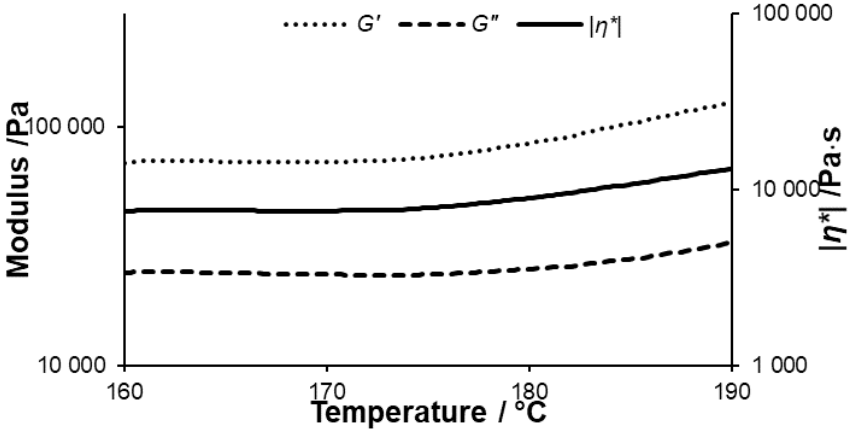

Figure S6. Temperature sweep from 150 to 190 °C of PAN sample F2 (8.1 mol-% DPA, $\overline{M}_n$ = 35, 000 g/mol, Đ = 5.3), mixed with 22.5 wt.-% PC. γ = 0.5 %, ω = 10 rad/s, heating rate = 1 K/min.

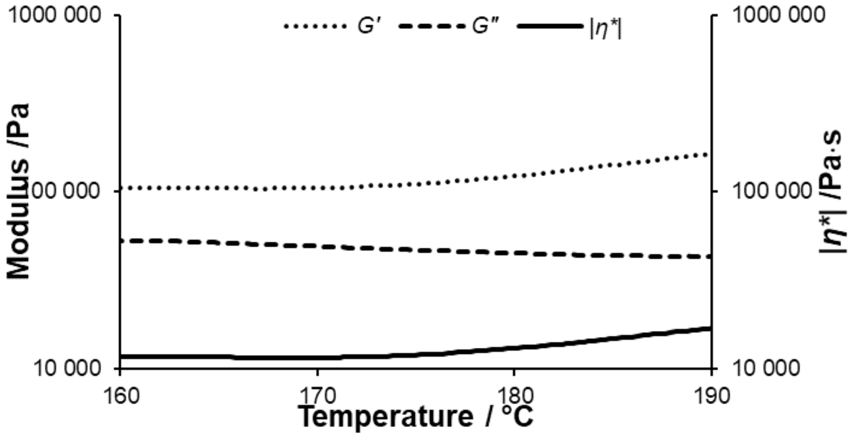

Figure S7. Temperature sweep from 150 to 190 °C of PAN sample F4 (6.1 mol-% DPA, 3.7 mol-% MA, $\overline{M}_n$ = 57, 000 g/mol, Đ = 5.5), mixed with 22.5 wt.-% PC. γ = 0.5 %, ω = 10 rad/s, heating rate = 1 K/min.

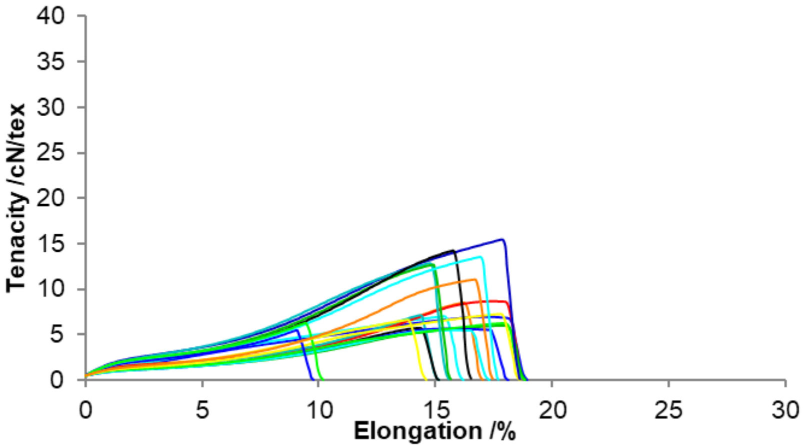

Figure S8. Stress-strain diagram (tensile test) of PAN F5 fibers (8.2 mol-% DPA, 3.8 mol-% MA, $\overline{M}_n$ = 43, 000 g/mol, Đ = 5.1) containing 22.5 wt.% PC wound at a winding speed of 30 m/min.

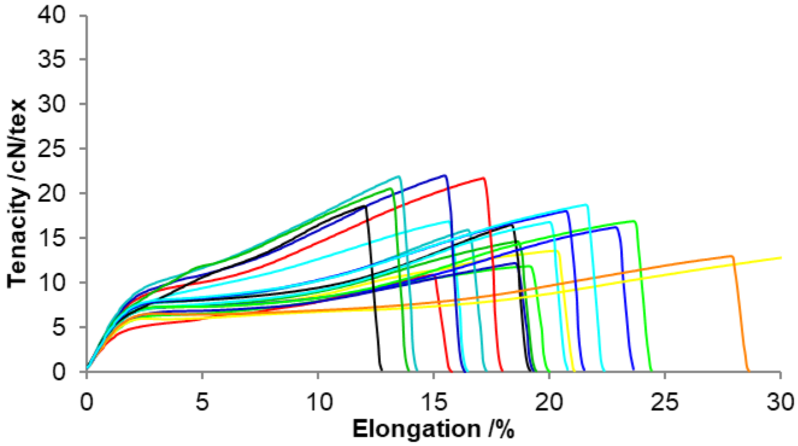

Figure S9. Stress-strain diagram (tensile test) of PAN F5 fibers spun (8.2 mol-% DPA, 3.8 mol-% MA, $\overline{M}_n$ = 43, 000 g/mol, Đ = 5.1), spun with 22.5 wt.% PC at a winding speed of 30 m/min. PC was washed out in demineralized water at 90 °C over 5 min.

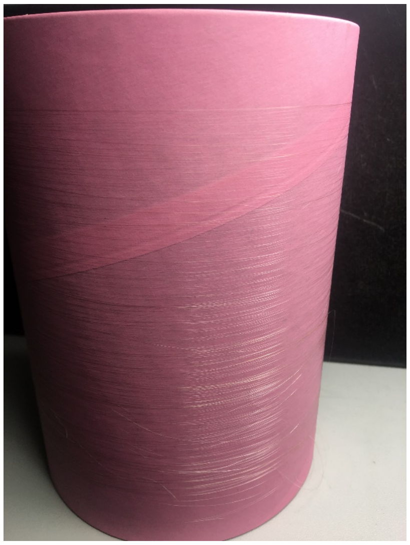

Figure S10. Photograph of PAN F5 (8.2 mol-% DPA, 3.8 mol-% MA, $\overline{M}_n$ = 43, 000 g/mol, Đ = 5.1) fibers containing 22.5 wt.% PC. Fibers were wound at a winding speed of 30 m/min.
